# Exploring the mechanism of the potential effects of Liujunzi Tang combined with Suanzaoren Tang in the treatment of lung cancer with insomnia based on network pharmacology and molecular docking technology

**DOI:** 10.1097/MD.0000000000042205

**Published:** 2025-04-18

**Authors:** Yaoyao Wang, Xin Zhao, Xiaomei Wang, Xuemei Wang, Tingting Zhang, Yanchun Wang

**Affiliations:** a Acupuncture and Moxibustion and Massage College of Henan University of Chinese Medicine, Zhengzhou, P.R. China; b Department of Chinese Medicine, Henan Provincial People’s Hosptial, People’s Hosptial of Zhengzhou University, Zhengzhou, P.R. China.

**Keywords:** insomnia, Liujunzi Tang combined with Suanzaoren Tang, lung cancer, molecular docking, network pharmacology

## Abstract

This study explores the potential mechanisms of Liujunzi Tang combined with Suanzaoren Tang in treating lung cancer with insomnia through network pharmacology and molecular docking methods. In the Traditional Chinese Medicine Systems Pharmacology (TCMSP) database, each single herb in the formula of Liujunzi Tang combined with Suanzaoren Tang was used as a search term, with repeated traditional Chinese medicines (TCMs) searched only once. Oral bioavailability (OB) ≥ 30% and drug-likeness (DL) ≥ 0.18 were set as screening criteria to obtain active components and targets of TCM. Disease-related targets for lung cancer with insomnia were obtained from the Gene Cards database, OMIM database, and PharmGKB database. The disease targets and drug effective component targets were intersected, and a disease-component intersection Venn diagram was drawn. The common intersection targets were imported into the DAVID database for Kyoto Encyclopedia of Genes and Genomics (KEGG) pathway and gene ontology (GO) function enrichment analysis, and molecular docking was performed to verify the affinity of active components with key targets. A total of 185 effective active components and 241 targets were obtained for Liujunzi Tang combined with Suanzaoren Tang. A total of 4290 disease targets were obtained from 3 disease databases. After removing 138 duplicate targets, a total of 4152 disease targets were obtained. The intersection of component targets and disease targets yielded 120 common targets. KEGG pathway enrichment was found in lipid and atherosclerosis, AGE-RAGE signaling pathway in diabetes complications, Kaposi sarcoma-related herpes virus infection, and other signaling pathways. Molecular docking showed that the core components naringenin had good binding activity with AKT1, quercetin and ginsenoside Rh2 with IL1B, and other targets. Through network pharmacology and molecular docking methods, it was explored that Liujunzi Tang combined with Suanzaoren Tang may treat lung cancer with insomnia through multiple components, multiple targets, and multiple pathways, regulating cancer pathways, lipid and atherosclerosis, and other signaling pathways, providing new ideas for the treatment of lung cancer with insomnia.

## 1. Introduction

Lung cancer is one of the most common cancers globally, and its incidence and mortality rates are also among the highest for cancers worldwide.^[1]^ A survey on the cancer epidemic situation between China and the United States shows that lung cancer has the highest mortality rate among all cancers, with lung cancer mortality accounting for approximately 28.49% of the total cancer mortality rate in China, and about 20.45% in the United States.^[2]^ Surveys and research predict that the incidence of lung cancer will continue to increase in most countries by 2035, posing a significant challenge to healthcare.^[3]^ Cancer can lead to the occurrence of insomnia, and insomnia is also a factor that increases the risk of cancer,^[4]^ in a study of 15,503 deaths due to sleep deprivation, lung cancer mortality accounted for about 10.2%,^[5]^ however, another study found that adequate sleep can help reduce the risk of lung cancer.^[6]^ Studies have shown that approximately 30% to 50% of lung cancer patients experience insomnia, with some studies reporting an incidence rate as high as 70%. Insomnia is more common in patients with advanced lung cancer. The occurrence of insomnia is closely related to factors such as the cancer itself, treatment side effects (e.g., chemotherapy, radiotherapy), psychological stress (e.g., anxiety, depression), and pain. Insomnia, in turn, leads to a decline in patients’ quality of life, impaired immune function, increased psychological burden, and reduced treatment adherence.^[7–9]^ Patients with lung cancer and insomnia have a lower quality of life, and to improve the quality of life of patients, it is urgent to address issues such as lung cancer with insomnia.

Traditional Chinese medicine (TCM) has the effect of reducing toxicity and enhancing efficacy. After targeted therapy for lung cancer patients, they are often mainly characterized by Qi deficiency or both Qi and Yin deficiency.^[10]^ Liujunzi Tang has the functions of invigorating Qi, strengthening the spleen, and drying dampness to transform phlegm, and it also has the characteristics of multi-target, multi-pathway, multi-channel, and multi-link treatment, which has an adjuvant therapeutic effect on cancer,^[11]^ and can also improve insomnia^[12]^; Suanzaoren Tang comes from “Synopsis of the Golden Chamber” and has the effects of nourishing blood, calming the mind, clearing heat, and relieving irritability. Pharmacological research suggests it has sedative-hypnotic, immune protection, and anti-inflammatory brain-protective effects,^[13]^ and it is often used clinically with syndrome differentiation and modification to treat various sleep disorders,^[14]^ and to clarify the specific mechanisms of treating lung cancer-related insomnia and to improve the efficacy of lung cancer-related insomnia and comprehensive treatment of lung cancer.^[15]^ The rationale for using combined TCM formulations to treat comorbid conditions lies in the theoretical foundation of TCM “treatment based on syndrome differentiation” and “holistic concept.” The combination of 2 formulations addresses both the pathogenesis of lung cancer and the symptoms of insomnia, reflecting the principles of treating both the root cause and the symptoms, as well as personalized treatment, in line with TCM comprehensive regulatory approach. Clinical findings have shown that the combination of Liu Jun Zi Tang combined with Suan Zao Ren Tang is highly effective in treating lung cancer-related insomnia.^[16–18]^ To clarify the multi-channel, multi-target action mechanism of the combined formula in treating lung cancer with insomnia, provide a reference basis for clinical application, and showcase the advantages of traditional Chinese medicine in disease treatment, network pharmacology is used to mine the effective components of the combined formula, and molecular docking technology is applied to verify the combination formula and the disease.

## 2. Materials and methods

### 2.1. Ingredient collection and screening

Using the Traditional Chinese Medicine System Pharmacology Database and Analysis Platform TCMSP database 2.3 (https://old.tcmsp-e.com/tcmsp.php), the Chinese medicines in the formula of Liujunzi Tang combined with Suanzaoren Tang were used as search terms, totaling 9 Chinese medicines, which are “suanzaoren,” “zhimu,” “fuling,” “chuanxiong,” “chenpi” and “gancaoe,” “renshen,” “banxia,” “baizhu” with oral bioavailability (OB) ≥ 30% and drug-likeness (DL) ≥ 0.18 as the screening criteria, the effective components of the combined formula were obtained. Through JSON, all targets of the Chinese medicine were accessed. Utilizing R language scripts, all targets corresponding to the effective components were obtained. Then, by downloading all human verified proteins from the UNIPROT database and using R language scripts, the genes corresponding to all targets of the effective components were acquired.

### 2.2. Lung cancer with insomnia disease target acquisition

By using the GeneCards database (https://www.genecards.org), OMIM database (https://www.omim.org), and PharmGKB database (https://www.pharmgkb.org), and using “lung cancer with insomnia” as the keyword, potential disease target genes related to lung cancer combined with insomnia were obtained. The data was then deduplicated using an Excel spreadsheet.

### 2.3. Drug-disease intersection targets and venn diagram mapping

The obtained drug active ingredient targets and disease genes were imported into the online creation of Venn diagram tool Venn 2.1.0 (https://www.bioinformatics.com.cn) to obtain the drug target and disease gene intersection target genes and to draw the Venn diagram.

### 2.4. Drug-disease protein–protein interaction network

Utilizing the STRING database (www.string-db.org), with “Multiple proteins” “Homo sapiens” “minimum required interaction score: high confidence (0.700)” “Network display options:hide disconnected nodes in the network” as the setting condition, and other parameters were kept unchanged in the initial setting. As the setting conditions, and other parameters remain unchanged, import the intersecting target genes in “List of Names” to obtain the drug-disease protein–protein interaction network diagram, and export the TSV format file; then use the Cytoscape 3.10.2 software and cytoNCA plug-in were used to determine the node Betweness, Closeness, Degree, Eigenvector, Local average connectivity, Local average connectivity, and Network as the main parameters, the TSV files obtained from STRING database were screened for PPI network graph topology to obtain the drug-disease core target genes, and molecular docking was performed on the core target genes.

### 2.5. GO biofunction and KEGG signaling pathway enrichment analysis

The obtained drug-disease intersection target genes were imported into the DAVID database (https://david.ncifcrf.gov) and searched by “Select Identifier:OFFICIAL_GENE_SYMBOL,” “Select species:Homo sapiens” “List Type:Gene List,” Gene Ontology (GO) analysis and Kyoto Encyclopedia of Genes and Genomics (KEGG) signaling pathway analysis were performed; selected *P* < .01 and the top 10 functions in the number of enriched genes were annotated,^[19]^ and the signaling pathways in KEGG signaling pathway with *P* < .01 and the top 25 enriched genes were selected, and the signaling pathways in the KEGG signaling pathway were annotated using the microbiology letter platform (www.bioinformatics.com.cn) to draw BP, CC, and MF triad two-sided bar graphs and Sankey bubble graphs for visualization and analysis respectively. Statistical test method: The hypergeometric test was used to evaluate the enrichment of target gene sets in GO terms and KEGG pathways. Multiple comparison correction: The Benjamini–Hochberg method was applied to control the false discovery rate (FDR). Significance level: A significance level of *P* < .05 was set, and terms with a corrected *P*-value (i.e., *q*-value) < .05 were considered significantly enriched.

### 2.6. Molecular docking

The core intersection target genes with the top 5 ranked degree values in the PPI network topology were used as receptors, and the active ingredients of drugs corresponding to the top 5 ranked core intersection target genes were used as small molecule ligands for molecular docking. Find the Entry ID corresponding to the core target genes in the UNIPORT database, then enter the protein data bank (PDB) database (https://www.rcsb.org), enter the Entry ID to search, find the 3D structure of the protein corresponding to the core target genes, download and save the PDB Format, and then import it into the PyMol-3.0.3 software, perform the Remove water molecules and impurities, and then save it in PDB format; import the active ingredients of drugs corresponding to the core target genes into PubChme database (https://pubchem.ncbi.nlm.nih.gov) to find the 2D results of small molecule ligands, download and save them in SDF format, and then import them into ChemBio3D Ultra14.0 software for optimization, obtain the 3D structure and save it in mol2 format; in AutoDockTools-1.5.7 software, import the protein receptor in PDB format, carry out hydrogenation and convert the format of the macromolecular receptor and save it in pdbqt format named “rep.pdbqt,”Then import the small molecule ligand named in mol2 format and save it in pdbqt format as “lig.pdbqt”; according to the action site of the protein receptor and the small molecule ligand, the center of the Grid Box and the length and width of the ligand, create the grid point of the protein receptor and determine the coordinates of the active pockets,^[20]^ save it in gpf format and name it as “grid.gpf,” run the obtained 3 files in Autodock_vina, import the obtained output.pdbqt file and rep.pdbqt file into PyMol-3.0.3 software for molecular docking, and select the docking result with the lowest free energy to save the picture.

## 3. Results and analysis

### 3.1. Results of screening of active ingredients and targets of Liujunzi Tang combined with Suanzaoren Tang

A total of 185 active ingredients were obtained from the screening of Liujunzi Tang combined with Suanzaoren Tang, including 9 active ingredients of suanzaoren, 7 active ingredients of chuanxiong, 5 active ingredients of chenpi, 13 active ingredients of banxia, 15 active ingredients of fuling, 92 active ingredients of gancao, 22 active ingredients of renshen, 7 active ingredients of baizhu, 15 active ingredients of zhimu, and the R language was utilized to obtain all the corresponding genes of the active ingredients by applying the UNIPROT database Using UNIPROT database and R language script to obtain the corresponding genes of all the targets of active ingredients, a total of 2206 targets were obtained, and 241 targets were finally obtained after removing 1965 duplicated targets.

### 3.2. Disease targets for lung cancer with insomni

Search for “lung cancer with insomnia” in the OMIM database, Gene Cards database, and PharmGKB database respectively, and a total of 4290 disease target genes were obtained. Among them, the OMIM database yielded 255 disease target genes, the Gene Cards database yielded 3751 disease target genes, and the PharmGKB database yielded 284 disease target genes. After deduplication using an Excel spreadsheet, a total of 4152 unique disease target genes were obtained.

### 3.3. Drug-disease intersection targets

**Figure 1.**
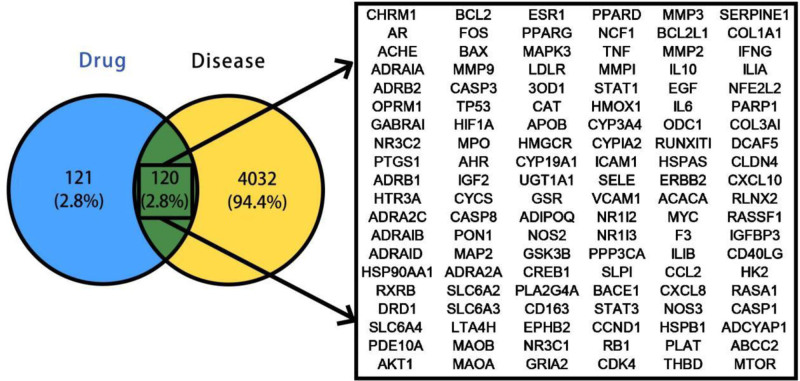
Liujunzi Tang combined with Suanzaoren Tang: Venn diagram of intersecting targets for lung cancer with insomnia. Blue represents Liu Jun Zi Tang combined with Suan Zao Ren Tang, and yellow represents lung cancer with insomnia.

The 241 effect target genes of Liujunzi Tang combined with Suanzaoren Tang and the 4152 disease target genes of Lung Cancer with Insomnia were imported into the online tool Venny 2.1.0 for making Venn diagrams, and the 2 were taken as intersections to obtain 120 intersecting target genes and to draw the Venn diagrams (Fig. 1).

### 3.4. Constructing a drug-disease protein–protein interaction network map

**Figure 2.**
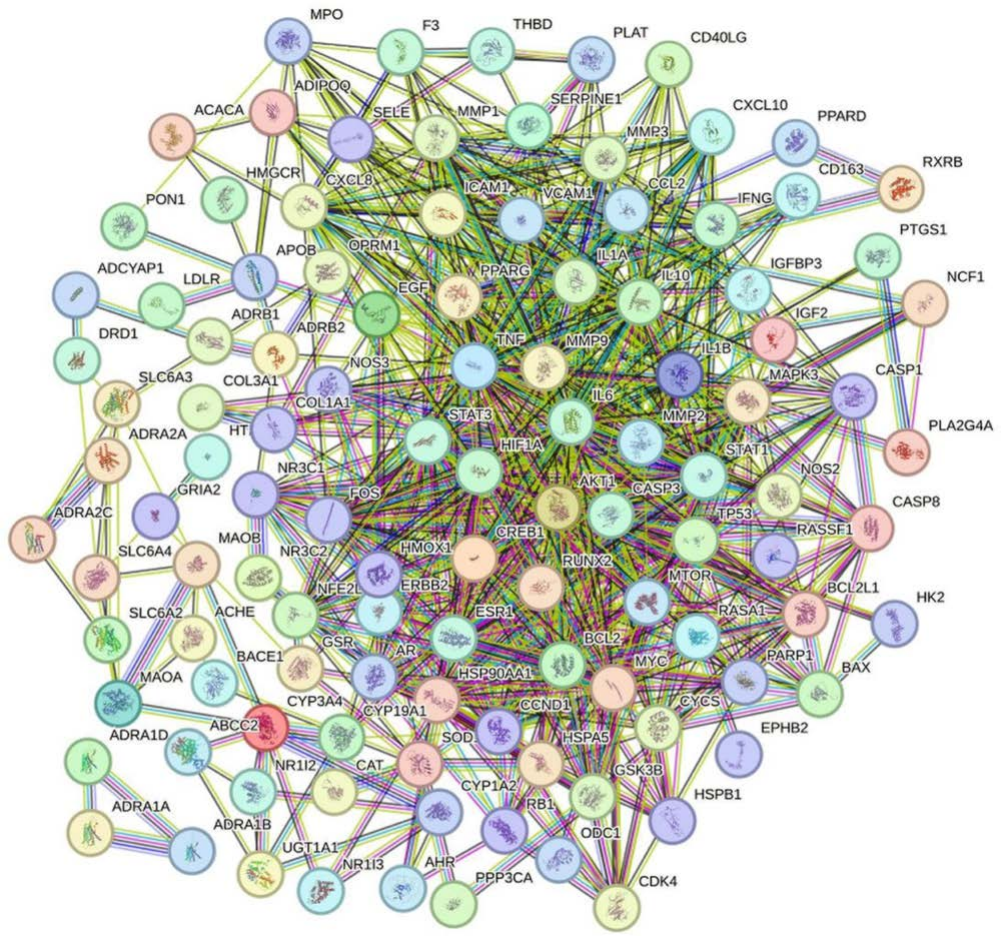
Protein interaction network diagram. PPI networks exported from the STRING database. PPI = protein–protein interaction,

**Figure 3.**
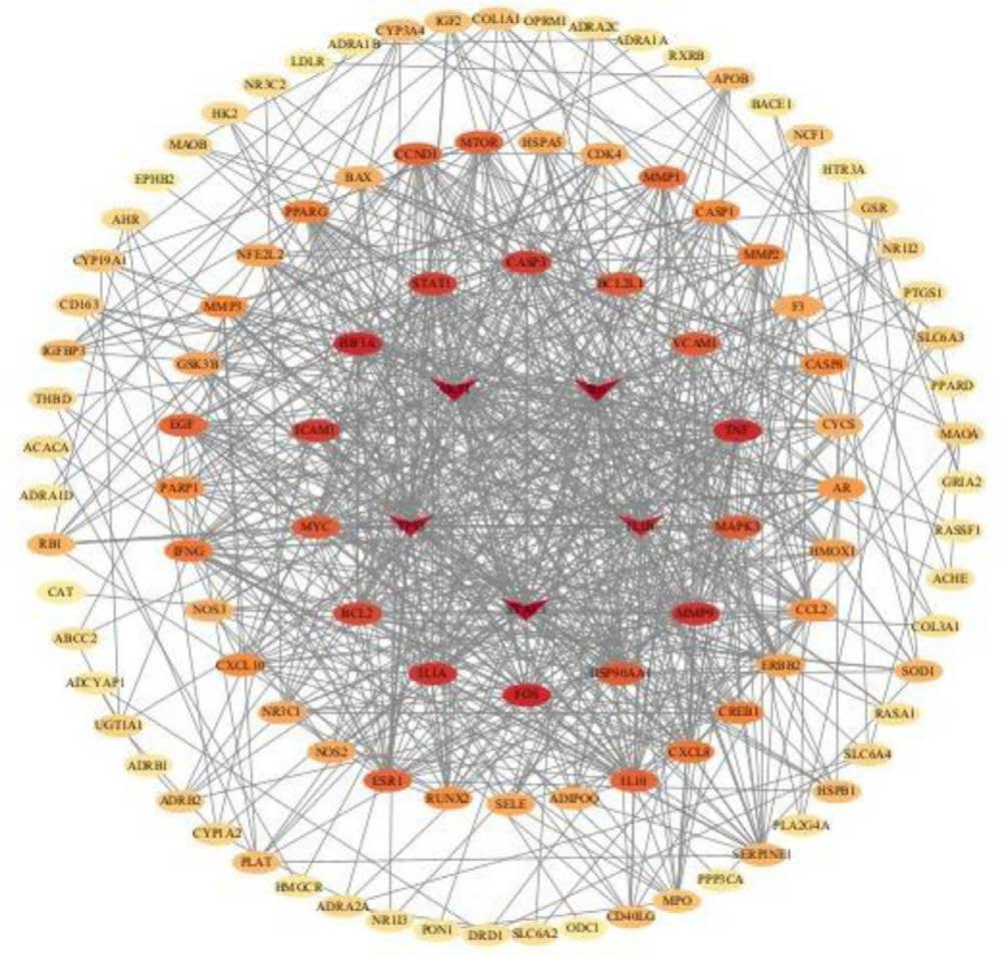
Core gene target screening. Using Cytoscape software to process the PPI network, the top 5 targets with the highest degree values are located at the center of the PPI network, including TP53, AKT1, IL6, IL1B, and STAT3.

The 120 intersecting target genes, imported into the STRING database, hid the disconnected nodes in the network, and selected the high confidence level (0.700) to construct the PPI network graph (Fig. 2), which contained 120 nodes, 748 edges, and an average node degree of 12.5; the TSV file obtained from the STRING database was imported into the Cytoscape 3.10.2 software, using cytoNCA plug-in for core gene screening to node parameters are greater than the central place value: Betweness 64.59300362,Closeness 0.180623974, Degree9, Eigenvector 0.049875759, Local average connectivity5.6, Network6.222222222, the first screening was performed, and 33 core genes were obtained: MMP2, HMOX1, CCL2, HSP90AA1, SERPINE1, ESR1, TP53, BCL2, EGF, GSK3B, NFE2L2, STAT3, CASP3, CYCS, AR, TNF, MAPK3, ERBB2, CXCL8, IL10, MMP9, MPO, RUNX2, AKT1, NR3C1, IFNG, IL6, MYC, PPARG, CCND1, HIF1A, IL1B, STAT1; with node parameters all greater than the central bit value: Betweness 8.901381745, Closeness 0.711111111, Degree19, Eigenvector 0.17462483, Local average connectivity13.78947368, Network16.02832168, a second screening was performed, and the 13 core genes were obtained: TNF, MAPK3, MMP9, TP53, AKT1 BCL2, IL6, MYC, HIF1A, IL1B, STAT1, STAT3, CASP3; to node parameters are greater than the central place value: Betweness 24.63555468, Closeness 0.820512821, Degree 25, Eigenvector 0.212222859, Local average connectivity 15.65217391, Network 21.77641023, the third screening was performed, and the 5 core genes were obtained: Tumor Protein p53 (TP53), AKT Serine/Threonine Kinase 1 (AKT1), interleukin 6 (IL6), Interleukin 1 Beta (IL1B), Signal Transducer and Activator of Transcription 3 (STAT3); the results are shown in Figure 3, from the outside to the inside, the deeper the color of the circle represents the greater the degree value of the core genes, and the 5 red triangles in the middle of the concentric circles are the core genes with the top 5 degree values, and the 5 core genes were finally subjected to molecular docking.

### 3.5. Constructing a component-disease visualization network diagram

**Figure 4.**
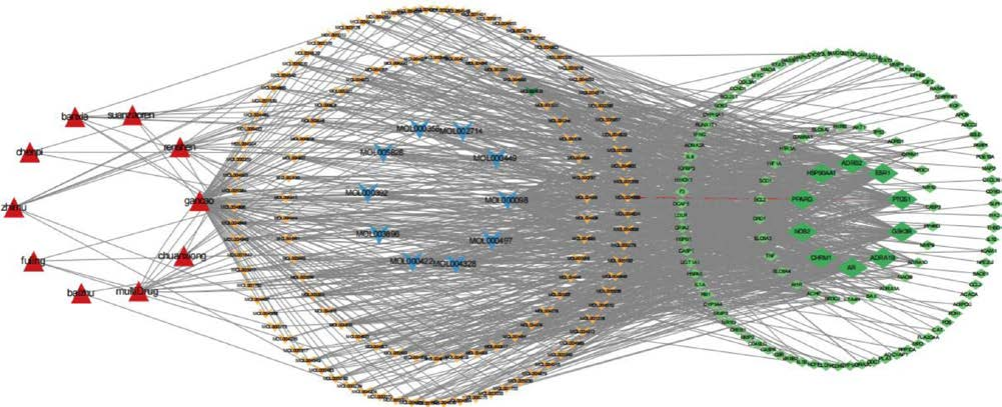
Component-Disease Regulatory Network Diagram. Red triangles, orange triangles, blue triangles in the middle, and green diamonds represent active components, target genes, and pathways, respectively. The larger the degree value, the darker the color and the larger the shape.

The component target gene file obtained from the R language script in 2.1 and the disease gene file after deduplication in 2.2 were run through the R language script to obtain the “net.network” file, “net.type” file, and “net.molLists” file, which were imported into Cytoscape 3.10.2 software for visual network diagram drawing, as shown in Figure 4, the leftmost red triangle circle represents the single medicine of liujunzi decoction combined with suanzaoren decoction, and there are 2 or more kinds of active ingredients, the more drug connections, the more active ingredients, and it can be seen that gancao has the most active ingredients; the middle triangular circle represents the active ingredients of the drug, the orange triangular circle from the outside to the inside, representing the degree value from small to large, the middle blue triangular circle is the top 10 active ingredients in the degree value ranking, the more lines in the figure, indicating that the active ingredients in the network the stronger the interactions; the rightmost green diamond-shaped circle is the intersection target gene of component-disease, from the outside to the inside, representing the degree value from small to large, and the large green diamond-shaped circle in the middle represents the intersection target gene of the top 10 in degree value, and the larger the diamond shape is, the stronger the correlation between the drug and the disease is; as can be seen in the figure, the effective active ingredient of each drug corresponds to more than one target gene, which is a more intuitive reflection of the multi-components, multi-targets, and multi-pathways of the treatment of lung cancer associated with insomnia in liujunzi decoction combined with suanzaoren decoction.

### 3.6. Results of GO biological function and KEGG signaling pathway enrichment analysis

**Figure 5.**
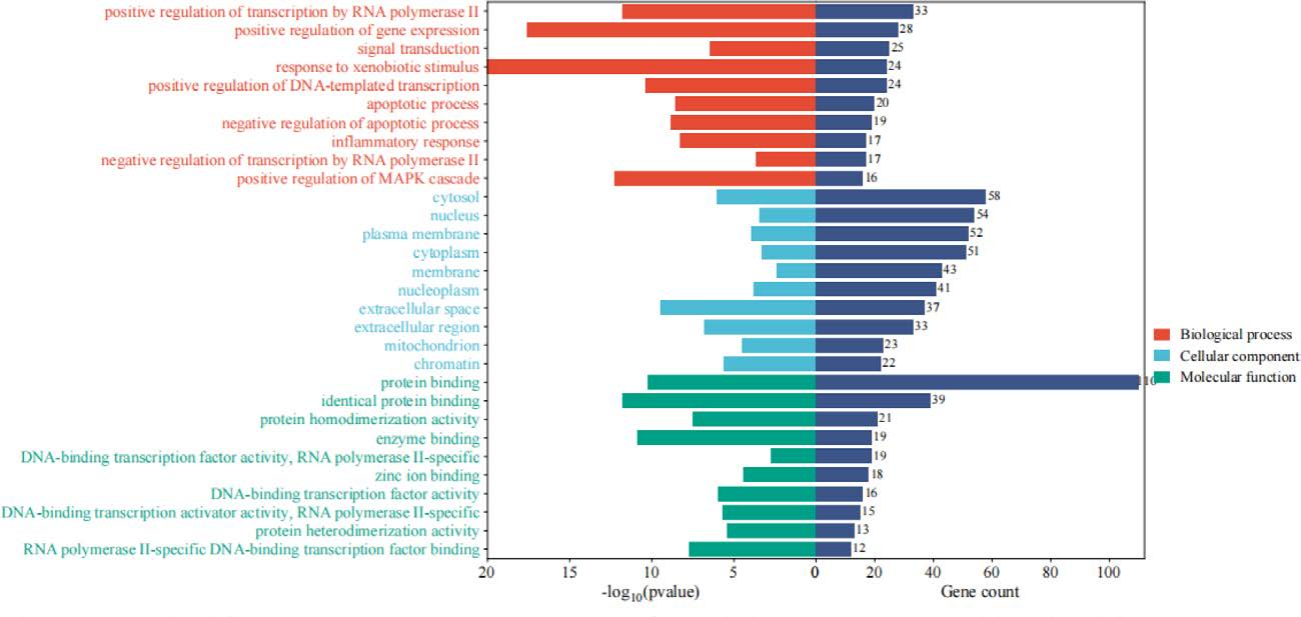
Significant GO enrichment terms for Liujunzi Tang combined with Suanzaoren Tang in the treatment of lung cancer with insomnia. Red = biological process, blue = cellular component, green = molecular function, GO = gene ontology.

The intersection target genes were imported into the DAVID database for GO and KEGG analysis, and in the GO molecular function enrichment analysis, a total of 748 enrichment results were obtained, of which 523 were biological processes (BP), 70 were cellular components (CC), and 155 were molecular functions (MF), and the above 3 parts of the *P* < .01, and the top 10 genes were ranked in the number of the functional annotations analyzed in the microbiological information platform, and plotted the BP, CC, and MF triad two-sided bar graphs (Fig. 5). The left side is the statistical value −log10(p); the right side is the number of enriched genes, and the results showed that GO functions were mainly enriched in positive regulation of gene expression, signal transduction, response to xenobiotic stimulus and other biological processes; cellular components such as cytosol and plasma membrane; and molecular functions such as protein binding and identical protein binding.

**Figure 6.**
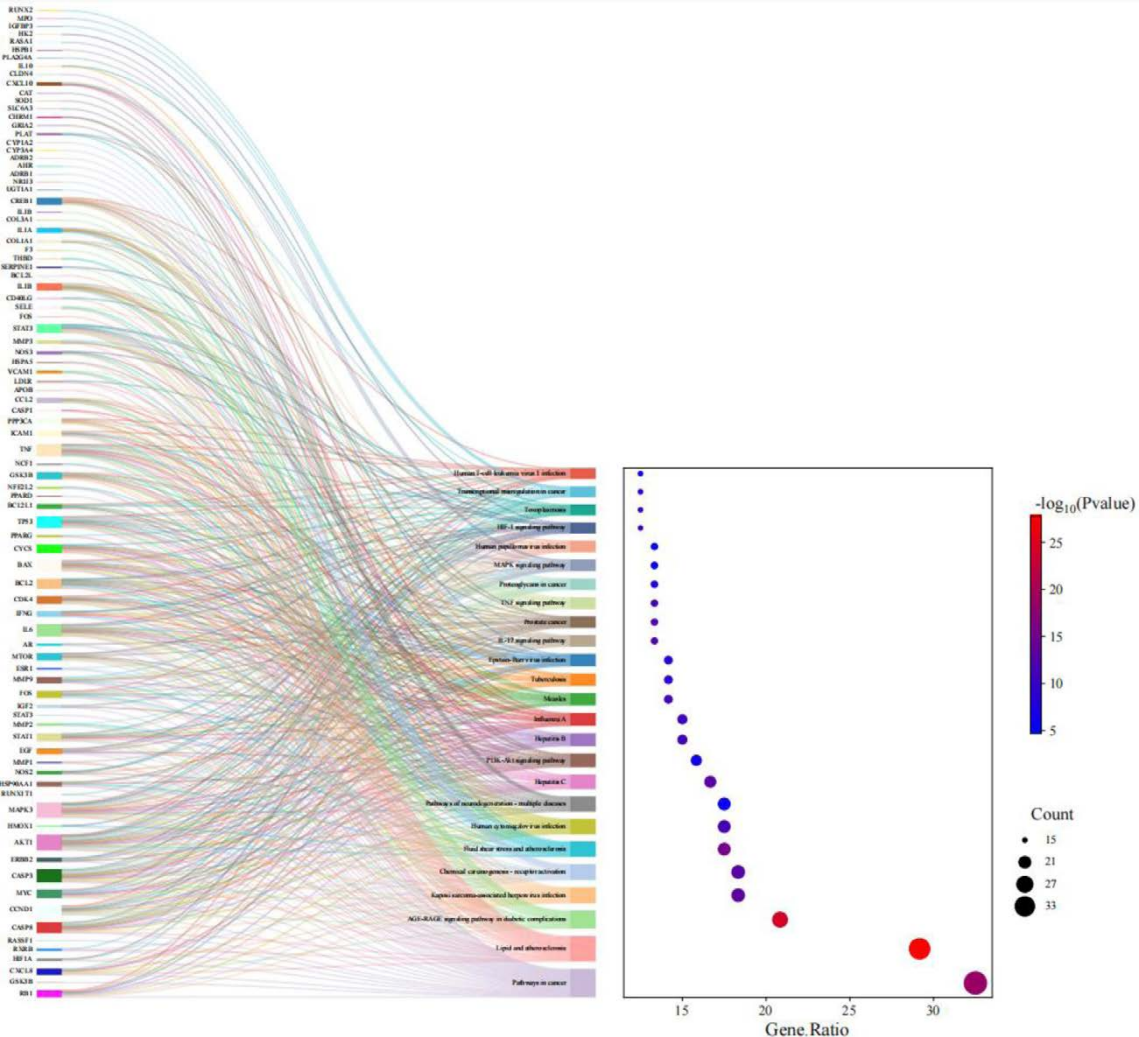
Significant KEGG enrichment terms for Liujunzi Tang combined with Suanzaoren Tang in the treatment of lung cancer with insomnia. KEGG = Kyoto Encyclopedia of Genes and Genomes pathway enrichment.

Among the KEGG signaling pathways, the pathways with *P* < .01 and the top 25 genes were selected for analysis, and the pathway-enriched Sankey bubble plots were drawn (Fig. 6). The left side represents the genes contained in each pathway, and the right side is a regular bubble map, the bubble size indicates the number of genes belonging to the pathway, and the bubble color indicates the *P*-value; the results showed that in the pathways in cancer, lipid and atherosclerosis, AGE-RAGE signaling pathway in diabetic complications, Kaposi sarcoma-associated herpesvirus infection, and many other signaling pathways.

### 3.7. Molecular docking results

The 5 core intersection target genes obtained were used as protein receptors to find their corresponding small molecule ligands (active ingredients), and the results showed that:the core gene AKT1 corresponded to the active ingredients baicalein, naringenin, kaempferol, quercetin, diosgenin,core gene IL1B corresponds to the active ingredient quercetin, ginsenoside Rh2, core gene IL6 corresponds to the active ingredient quercetin, core gene TP53 corresponds to the active ingredients baicalein, quercetin, diosgenin, and nobiletin, and the core gene STAT3 corresponded to licochalcone A.

Using molecular docking technology, each core gene and the active ingredient were docked separately, and the docking binding energy results showed that (Table 1): the docking binding energy of core gene AKT1 and the active ingredient baicalein was lower than −6.8 kcal/mol, and that of naringenin was lower than −7.4 kcal/mol, kaempferol docking binding energy is lower than −6.1 kcal/mol, quercetin docking binding energy is lower than −6.3 kcal/mol, diosgenin docking binding energy was lower than −8.2 kcal/mol. The docking binding energies of the core gene IL1B and the active ingredient quercetin and ginsenoside Rh2 were lower than −7.3 kcal/mol. The docking binding energies of the core gene IL6 with the active ingredient quercetin were lower than −7.3 kcal/mol. The docking binding energies of the core gene TP53 with the active ingredient baicalein and diosgenin and nobiletin were lower than −9.4 kcal/mol, quercetin was lower than −7.5 kcal/mol. The core gene STAT3 docking binding energy with the active ingredient licochalcone a was lower than −7.7 kcal/mol.

**Table 1:** Results of docking between core genes and corresponding active component molecules.

Core gene	Uniprot ID	PDB ID	Active ingredient	Docking binding energy (kcal/mol)
AKT1	P31749	1H10	Baicalein	−6.8
			Naringenin	−7.4
			Kaempferol	−6.1
			Quercetin	−6.3
			Diosgenin	−8.2
IL1B	P01584	1HIB	Quercetin	−7.3
			Ginsenoside Rh2	−7.3
IL6	P05231	1ALU	Quercetin	−6.9
TP53	P04637	1DT7	Baicalein	−9.4
			Quercetin	−7.5
			Diosgenin	−9.4
			Nobiletin	−9.4
STAT3	P40763	6NJS	Licochalcone A	−7.7

Note: By calculating the energy contributions of weak interactions such as hydrogen bonds and van der Waals forces, and integrating them into the Gibbs free energy formula (∆G = ∆H−T ∆ S), the binding stability can be assessed. A lower energy value (typically represented as a negative value) indicates a stronger binding affinity.

By calculating the energy contributions of weak interactions such as hydrogen bonds and van der Waals forces, and integrating the Gibbs free energy formula (∆G=∆H-T ∆ S) to evaluate binding stability, lower energy values (typically represented as negative values) indicate stronger binding. Generally, a binding energy below −4.25 kcal/mol suggests that a small molecule has specific binding activity to a protein, below −5.0 kcal/mol indicates good binding activity, and below −7.0 kcal/mol signifies strong binding activity. The binding energies between the small molecule ligands and protein receptors are all less than −5 kcal/mol, indicating favorable binding affinity between the molecules and proteins.

**Figure 7.**
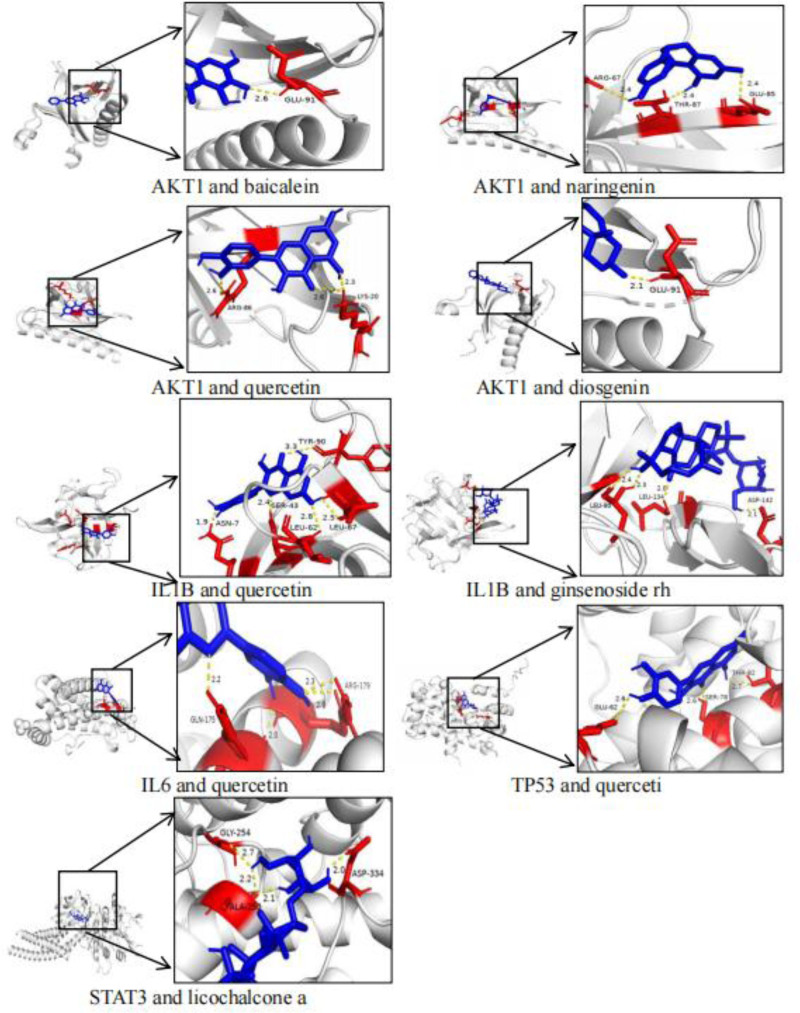
Molecular docking model diagram, selecting small molecule ligands with strong binding affinity to protein receptors for molecular docking. The selected pairs are AKT1 with baicalein, naringenin, quercetin, and diosgenin; IL1B with quercetin and ginsenoside Rh2; IL6 with quercetin; TP53 with quercetin; and STAT3 with licochalcone A.

Constructing the molecular docking model of core genes and active ingredients (Fig. 7), the results showed that AKT1 formed hydrogen bonding with baicalein at GLU-91 site, with naringenin at ARG-67, THR-87, GLU-85 sites, with quercetin at LYS-20, ARG-86 sites, and with diosgenin element at GLU-91 site but kaempferol no hydrogen bonding sites are formed; IL1B formed hydrogen bonds with quercetin at sites TYR-90, ASN-7, SER-43, LEU-62, LEU-67, and ginsenoside Rh2 at sites ASP-415, LEU-134, and LEU-80; IL6 formed hydrogen bonds with quercetin at sites GLN-175 and ARG-179; and TP53 formed hydrogen bonds with quercetin at THK-82, SER-78, and GLU-62, but not with baicalein, diosgenin and nobiletin; STAT3 formed hydrogen bonds with licochalcone a at GYL-254, ALA-250, and ASP-334 sites.

## 4. Discussion

Insomnia not only affects the quality of life of lung cancer patients and increases their pain, but also increases the overall rate of lung cancer, and also increases the risk of lung adenocarcinoma and squamous cell lung cancer.^[21–24]^ Lung cancer causes insomnia, and insomnia further increases pain, anxiety and other symptoms. There is a strong association between insomnia and lung cancer, and the 2 affect each other,^[25]^ so in order to improve the health and quality of life of patients and reduce pain, it is urgent to solve the problems of lung cancer and insomnia.

Network pharmacology was utilized to obtain the targets of Liujunzi Tang combined with Suanzaoren Tang for the treatment of lung cancer with insomnia, in which the main components are quercetin, baicalin, diosgenin, licochalcone a, and nobiletin, among others. Some studies have proved that quercetin inhibits cellular pyroptosis and reduces cellular inflammatory injury through STAT3 pathway,^[26]^ exerts anticancer effects by inhibiting PI3K/Akt/GSK-3β signaling pathway^[27]^ and inhibits proliferation of A549 cells and induces iron death, which in turn leads to cell apoptosis.^[28]^ Baicalein can inhibit the proliferation, migration and invasion of non-small cell lung cancer cells, promote their apoptosis, and regulate the biological behavior of non-small cell lung cancer cells through the miR-424-3p/PTEN/PI3K/AKT pathway.^[29]^ There are also studies that show that baicalein can inhibit the growth of A549 cells, and block the growth cycle of the cells, thereby inducing cell death.^[30]^ Some studies have shown that Diosgenin plays an anticancer role by inhibiting metastasis, proliferation, growth, and inducing apoptosis of lung cancer H1650 cells,^[31,32]^ and also has in vitro antitumor activity.^[33]^ Licochalcone a can inhibit the activation of NF-κB/STAT3 signaling pathway, and then inhibit the expression of related inflammatory factors, can also inhibit cell growth and ultimately lead to cell apoptosis.^[34,35]^ It has been shown that nobiletin can inhibit the proliferation of cancer cells both in vivo and in vitro, and induce apoptosis in non-small cell lung cancer A549 cells.^[36,37]^

In this study, we screened 5 core target genes, namely AKT1, IL1B, IL6, TP53, and STAT3. Akt1 is a serine/threonine protein kinase, which acts as a proto-oncogene and is involved in the regulation of cellular metabolism, growth, and development. It has been demonstrated that the selective AKT1 inhibitor, A-674563, is a more potent regulator of cellular survival, and that, in the treatment of lung cancer, the Compounds selectively targeting AKT1 are more effective than compounds of the 3 isoforms of AKT.^[38,39]^ Interleukin 1β, a pro-inflammatory factor, is closely related to cancer and can affect apoptosis and DNA pathway repair, and interleukin 1β is elevated when infected.^[40,41]^ IL6 also acts as a pro-inflammatory factor and induces the differentiation and proliferation of non-small-cell lung cancer cells neuroendocrine cells^.[42]^ It has been demonstrated that the concentration of IL6 increases linearly in NSCLC tumor staging from early to advanced stages and is lost. The concentration of IL6 increases linearly from early to advanced stages of NSCLC tumors, and IL6 is also elevated during insomnia.^[43,44]^ STAT3 (signal transducer activator of transcription 3), as a key node in the signaling pathway of many solid tumors, is resistant to tumors, and the blockade of the STAT3 gene effectively inhibits the expression of relevant drug-resistant proteins in the signaling pathway, thus improving the effect of antitumor therapy.^[45]^ Similarly, some studies have proved that cytokines secreted by TAMs can activate the JAK2/STAT3 signaling pathway in NSCLC cells, thus inhibiting the apoptosis of NSCLC cells.^[46]^ As a kind of tumor suppressor, the activation of TP53 protein can lead to a variety of cellular responses, including apoptosis, cell senescence, cell cycle blockade, DNA repair, cell death, and cell senescence, cell cycle arrest, DNA repair, metabolic adaptation, and alteration of cellular properties, which is important for tumor suppression.^[47]^

Through molecular docking, it was found that AKT1 had good binding properties with baicalein, kaempferol, naringenin and quercetin; IL1B with quercetin and ginsenoside Rh2; IL6 with quercetin; TP53 with quercetin; and STAT3 with licochalcone a all have good binding properties. This suggests that the liujunzi decoction combine with suanzaoren decoction may exert its therapeutic effect in the treatment of lung cancer with insomnia by affecting the expression of AKT1, IL1B, IL6, TP53, STAT3 to play a role in the treatment of lung cancer with insomnia. GO and KEGG analyses revealed that pathways in cancer, lipid and atherosclerosis, AGE-RAGE signaling pathway in diabetic complications signaling pathway were most strongly associated with lung cancer with insomnia and were more enriched, which were likely to be the potential mechanisms and pathways for the treatment of lung cancer with insomnia with the Liujunzi Tang combined with Suanzaoren Tang. In the pathways in cancer, lipid and atherosclerosis, AGE-RAGE signaling pathway in diabetic complications signaling pathway, a large number of gene targets related to the present study were found, especially AKT1, IL1B, IL6, TP53, and STAT3, which accounted for an important part of the signaling pathway, so it can be seen that Liujunzi Tang combined with Suanzaoren Tang are very likely to exert therapeutic effects by inhibiting the proliferation of cancer cells, blocking the growth cycle of cancer cells, and inducing apoptosis of cancer cells through the role pathway of pathway in cancer, lipid and atherosclerosis pathway, and AGE-RAGE signaling pathway in diabetes complications.

This study has some limitations. Firstly, data dependency and quality limitations: This study heavily relies on the quality and completeness of bioinformatics databases. If the data contains errors, omissions, or inaccuracies, it may affect the accuracy of the constructed network models, leading to incorrect conclusions. Additionally, if the databases are not updated in a timely manner, the study results may also be biased. Secondly, limitations of static models: Biological systems are dynamic, whereas network pharmacology models are typically constructed based on static data, making it difficult to accurately reflect the dynamic changes in biomolecular networks under different physiological and pathological conditions. This limitation of static models may result in discrepancies between the study results and the complexity of real biological systems. Lastly, approximations in molecular docking and challenges in experimental validation: The force fields and algorithms used in molecular docking involve certain approximations in describing molecular interactions and cannot precisely simulate all physicochemical processes (such as solvent effects and induced fit), which may lead to inaccuracies in docking results. Furthermore, molecular docking is a computer-simulated validation method. While it can provide preliminary predictive results, its accuracy requires extensive experimental validation. However, experiments are often challenging, and the simulation results may not be fully verified. Therefore, in the future, it is essential to closely integrate molecular docking results with experimental data, using experimental validation to optimize docking models and parameters, while guiding docking research with experimental outcomes to enhance the reliability of predictions. Additionally, artificial intelligence and machine learning technologies can be leveraged to train models using large volumes of experimental data, improving the predictive capability of molecular interactions. In clinical settings, the designed compounds can be synthesized and subjected to activity screening, with multiple rounds of optimization to enhance drug-like properties, including stability, solubility, and bioavailability. Furthermore, the potential of combining this drug with existing clinical medications can be explored to increase therapeutic efficacy. Despite these considerations, the results of this study provide a commendable direction for the design of our experimental endeavors.

## 5. Conclusions

To sum up, the main active ingredients of Liujunzi Tang combined with Suanzaoren Tang, such as quercetin, baicalin, diosgenin, licochalcone a, and others, may treat lung cancer with insomnia through disease targets such as AKT1, IL1B, IL6, TP53, and STAT3, and specifically may work through the pathways in cancer, lipid and atherosclerosis, AGE-RAGE signaling pathway in diabetic complications and other signaling pathways, which play a role by participating in the inhibition of cancer cell proliferation, blocking the growth cycle of cancer cells, and inducing apoptosis of cancer cells, which provides a certain basis for further research.

## Author contributions

**Conceptualization:** Xiaomei Wang.

**Data curation:** Yaoyao Wang, Xuemei Wang.

**Funding acquisition:** Yanchun Wang.

**Methodology:** Yaoyao Wang.

**Project administration:** Tingting Zhang.

**Resources:** Yanchun Wang.

**Software:** Xin Zhao.

**Supervision:** Xuemei Wang, Tingting Zhang.

**Visualization:** Yanchun Wang.

**Writing – original draft:** Yaoyao Wang, Xin Zhao, Xiaomei Wang.

**Writing – review & editing:** Yanchun Wang.
